# Alstonine as an Antipsychotic: Effects on Brain Amines and Metabolic Changes

**DOI:** 10.1093/ecam/nep002

**Published:** 2011-06-23

**Authors:** Viviane M. Linck, Ana P. Herrmann, Ângelo L. Piato, Bernardo C. Detanico, Micheli Figueiró, Jorge Flório, Maurice M. Iwu, Christopher O. Okunji, Mirna B. Leal, Elaine Elisabetsky

**Affiliations:** ^1^Laboratório de Etnofamacologia, ICBS, Universidade Federal do Rio Grande do Sul, Rua Sarmento Leite 500/202, 90050-170 Porto Alegre, RS, Brazil; ^2^Programa de Pós-Graduação em Ciências Farmacêuticas, UFRGS, Porto Alegre, Brazil. Av. Ipiranga, 2752, 1° andar, 90610-000 Porto Alegre, RS, Brazil; ^3^Departamento de Patologia, Faculdade de Medicina Veterinária e Zootecnia, Universidade de São Paulo, USP, São Paulo-SP 05508-900, Brazil; ^4^International Centre for Ethnomedicine and Drug Development, University of Nigeria, Nsukka, Nigeria; ^5^Bioresources Development and Conservation Programme, University of Nigeria, Nsukka, Nigeria

## Abstract

Managing schizophrenia has never been a trivial matter. Furthermore, while classical antipsychotics induce extrapyramidal side effects and hyperprolactinaemia, atypical antipsychotics lead to diabetes, hyperlipidaemia, and weight gain. Moreover, even with newer drugs, a sizable proportion of patients do not show significant improvement. Alstonine is an indole alkaloid identified as the major component of a plant-based remedy used in Nigeria to treat the mentally ill. Alstonine presents a clear antipsychotic profile in rodents, apparently with differential effects in distinct dopaminergic pathways. The aim of this study was to complement the antipsychotic profile of alstonine, verifying its effects on brain amines in mouse frontal cortex and striatum. Additionally, we examined if alstonine induces some hormonal and metabolic changes common to antipsychotics. HPLC data reveal that alstonine increases serotonergic transmission and increases intraneuronal dopamine catabolism. In relation to possible side effects, preliminary data suggest that alstonine does not affect prolactin levels, does not induce gains in body weight, but prevents the expected fasting-induced decrease in glucose levels. Overall, this study reinforces the proposal that alstonine is a potential innovative antipsychotic, and that a comprehensive understanding of its neurochemical basis may open new avenues to developing newer antipsychotic medications.

## 1. Introduction

The management of schizophrenic symptoms has never been a trivial matter, further complicated by the low adherence to treatments and the serious side effects of available drugs. Classical antipsychotics, blocking D_2_ dopamine receptors, lead to extrapyramidal effects related to antagonism in the nigrostriatal pathway [1], and hyperprolactinaemia due to antagonism in the tuberoinfundibular pathway [2]. In the early 1990s a new class of antipsychotics was introduced in the clinic, with the alleged advantage of causing no or minimal extrapyramidal (EPS) side effects [3, 4], and the resulting potential to increase treatment adherence. Unfortunately, however, it is now recognized that atypical antipsychotics often induce diabetes, hyperlipidaemia and weight gain [5–7]. For instance, patients on clozapine have a 4 times higher chance of developing diabetes, hyperglycaemia, and hyperlipidaemia than patients on classical antipsychotics [8]. The mechanisms by which these metabolic alterations are produced are not entirely understood [9].

Post marketing surveillance has shown that the so-called atypical antipsychotic drugs are indeed quite heterogeneous pharmacologically, varying in effectiveness, as well as metabolic and EPS side effects [10]. It is thought that atypical antipsychotics fail to induce significant EPS effects due to their weakened blockade of D_2_ dopamine (DA) receptors combined with interactions with various neurotransmitters, especially serotonin (5HT) and in particular the 5HT_1A_, 5HT_2A_ and 5HT_2C_ receptors [1]. Accordingly, specific DA pathways may be modulated by serotonin receptors, depending on the presence of serotonergic receptor sub-types [11]. Given that positive and negative symptoms of schizophrenia are thought to reflect an imbalance in DA mesolimbic and mesocortical pathways [12], and the problems seen with merely blocking DA receptors, it is an attractive idea that 5HT receptors can precisely modulate different DA pathways.

**Figure 1:**
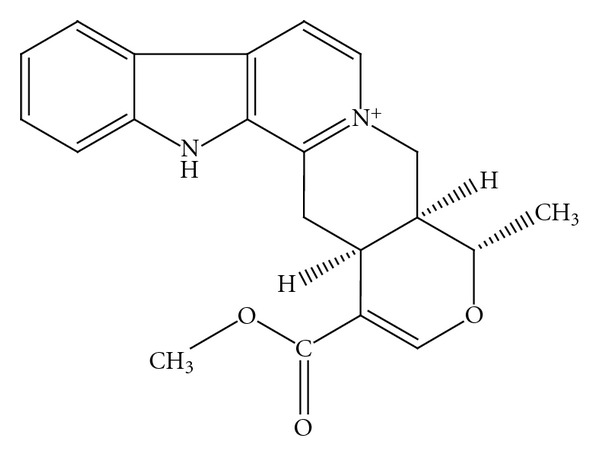
Alstonine.

The indole alkaloid alstonine (Figure 1) was identified as the major component of a plant-based treatment given to mentally ill patients in Nigeria [13]. Alstonine shows a clear antipsychotic profile in rodents, closer to atypical than to classical agents [14]. Apparently, alstonine induces dissimilar effects in dopaminergic pathways: while apomorphine-induced stereotypy and amphetamine-induced lethality were significantly reduced by alstonine, suggesting a decrease in mesolimbic DA, alstonine reversed haloperidol-induced catalepsy, indicating that nigrostriatal dopamine transmission is not lessened [14]. Relevant for the treatment of negative symptoms, alstonine also presents anxiolytic properties, involving 5HT_2A,C_ receptors, and reverses interaction deficits induced by MK801 [15, 16].

In order to better understand the basis of alstonine's antipsychotic profile, the aim of this study was to verify alstonine effects on brain amines in mouse frontal cortex and striatum. Additionally, we examined if alstonine causes the same hormonal and metabolic changes induced by classical and atypical antipsychotics.

## 2. Materials and Methods

### 2.1. Plant Material

Mature fruits of *Picralima nitida* Stampf Th. et H.Dur. were collected by staff from the International Centre of Ethnomedicine and Drug Development (InterCEDD) in February and March 2002, in Nnewi, Anambra state, Nigeria. The plant material was identified by comparison with a voucher specimen (UNN/83/07) at the Department of Pharmacognosy Herbarium of the University of Nigeria, Nsukka, and authenticated by Mr A. Ozioko of the Department of Botany of the same university. The fruit rind was separated, dried, and pulverized. The powdered material was successively extracted with n-hexane, methylene chloride, and methanol. Extracts were concentrated under vacuum using a rotary evaporator.

### 2.2. Isolation and Identification of Alstonine

Pure alstonine hydrochloride used for this investigation was isolated from the fruit rind of *P. nitida* Stampf Th. et H.Dur. (Apocynaceae). The separations were performed using pH-zone-refining counter-current chromatography as previously described [17]. Briefly, the experiment was performed with a two-phase solvent system composed of methyl tert-butyl ether (MtBE)-acetonitrile-water (2 : 2 : 3, v/v), where triethylamine (TEA) was added to the upper organic stationary phase as a retainer, and hydrochloric acid (HCl) to the aqueous mobile phase as an eluter. The basic organic phase was used as the stationary phase and the acidic lower phase was used as the mobile phase. The separation was initiated by filling the entire column with the stationary phase using the LC pump, and then loading the sample. The sample solution was prepared by dissolving 15.0 g of alkaloid fraction of the methylene chloride extract of *P. nitida* in 100 mL of a phase mixture consisting of equal volumes of each phase. The mobile phase was then pumped into the column at 2 mL min^−1^ while the column was rotated at 834 rpm in the combined head to tail elution mode [18, 19]. The absorbance of the eluate was continuously monitored at 280 nm and 4 mL fractions were collected. The pH of each eluted fraction was measured with a pH meter and fractions were dried using a Speed Vac. Identification of pH-zone refining counter-current chromatography pure fractions was carried out by using thermospray liquid chromatography-mass spectrometry (LC-MS) and by TLC co-elution experiments with reference alstonine samples provided by InterCEDD, Nsukka, Nigeria.

#### 2.2.1. Drugs

Clozapine was purchased from Sigma Chemical Co. (St Louis, MO, US), and haloperidol was used as commercial Haldol (Janssen Farmacêutica Ltda, SP, Brazil). Clozapine was solubilized in HCl (0.1N) and its pH adjusted to 6.0 with NaOH 0.5N; alstonine and haloperidol were diluted in water. All drugs were administered intraperitoneally (ip) in a volume of 0.1 mL/10 g of body weight.

#### 2.2.2. Animals

Experiments were performed using male (CF1) adult albino mice, received from Fundação Estadual de Produção e Pesquisa em Saúde (FEPPS) at 2 months of age (40–45 g). Animals were maintained in our own animal facility under controlled environmental conditions (22 ± 1°C, 12 h-light/dark cycle, free access to food (Nuvilab CR1) and water), for at least 2 weeks before the experiments.

The project was approved by the University ethics committee (approval #2007706) and all procedures were carried out in accordance with institutional policies on the handling of experimental animals.

#### 2.2.3. Brain Amines HPLC-ED Determination

Animals (*n* = 10) received ip saline or alstonine 1.0 mg kg^−1^, and 30 min later were sacrificed by decapitation. Brains were rapidly removed and dissected over dry ice; striatum and frontal cortex were removed, weighed and immediately stored in liquid nitrogen. Not later than a week following sample collections, tissues were homogenized in 0.1 M perchloric acid by manual sonication. DA and its metabolites dihydroxyphenylacetic acid (DOPAC) and homovanillic acid (HVA), as well as 5-HT and its metabolite 5-hydroxyindole acetic acid (5-HIAA) were measured by reverse-phase high performance liquid chromatography (HPLC) with electrochemical detection, as described by Felicio et al. [20]. Briefly, the HPLC-ED system (model 6A; Shimatzu, Kyoto, Japan) consisted of a C-18 column (Shimpak; ODS, Kyoto, Japan), an electrochemical detector (model 6A; Shimatzu), a sample injector (valve for 20 *μ*l), and an integrator (model 6A Chromatopac; Shimatzu). Dihydroxybenzylamine (DHBA) was used as the internal standard, and peak areas of external standard were used to quantify the sample peaks. The time for each sample was 28 min. Values are expressed as ng/g tissue weight. The detection limit was 0.002 ng for DA, DOPAC, 5-HT, and 5-HIAA, and 0.02 ng for HVA.

#### 2.2.4. Glycaemia

Experiments were conducted between 9 a.m. and 12 midday. Groups of mice (*n* = 5–9) were treated ip with saline, clozapine (2.0 and 10.0 mg kg^−1^), and alstonine (0.5 and 1.0 mg kg^−1^). Food and water were withdrawn immediately before treatments and glucose measurements were taken before and 3 hours after drug administration [7], with a glucometer (Accu-Chek Active Roche), using a blood drop drawn from the tail.

#### 2.2.5. Body Weight

Mice (*n* = 8–10) were treated ip for 6 days with saline, haloperidol (0.25 mg kg^−1^), clozapine (2.0 mg kg^−1^) or alstonine (0.5 and 1.0 mg kg^−1^). The body weight was measured daily between 12 a.m and 1 p.m.

#### 2.2.6. Prolactin (PRL) Levels

Groups of mice (*n* = 6–8) were treated with ip injection of saline, haloperidol (0.25 mg kg^−1^), clozapine (2.0 mg kg^−1^) or alstonine (1.0 mg kg^−1^). Animals were decapitated 30 minutes after treatments; the blood was collected and centrifuged for 10 minutes at 5000 rpm and serum was stored at −20°C until assayed. Plasma PRL levels were measured in duplicate by radioimmunoassay (RIA), using materials provided by NIADDK (Bethesda, MD, USA). The intra- and inter-assay coefficients of variation were 6% and 8%, respectively [21].

#### 2.2.7. Statistical Analysis

HPLC data were evaluated by independent *t*-test. Group differences in glucose and prolactin levels were analyzed by one-way ANOVA, followed by Duncan; pre and post drug glucose levels were analyzed by paired *t*-test. ANOVA with repeated measures followed by Duncan was used to evaluate weight gain. *P*-value <.05 was considered significant.

## 3. Results

**Figure 2:**
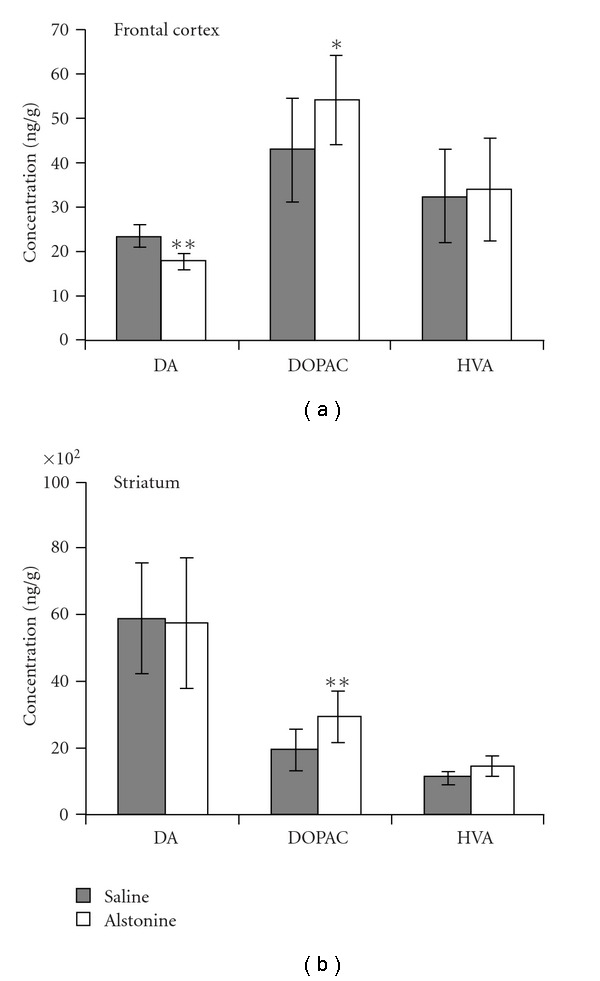
Dopamine (DA) and its metabolites (DOPAC and HVA) in mouse frontal cortex (a) and in striatum (b) Mean ± SD. *P < .05, **P < .01 when compared with saline, Independent *t*-test.

**Figure 3:**
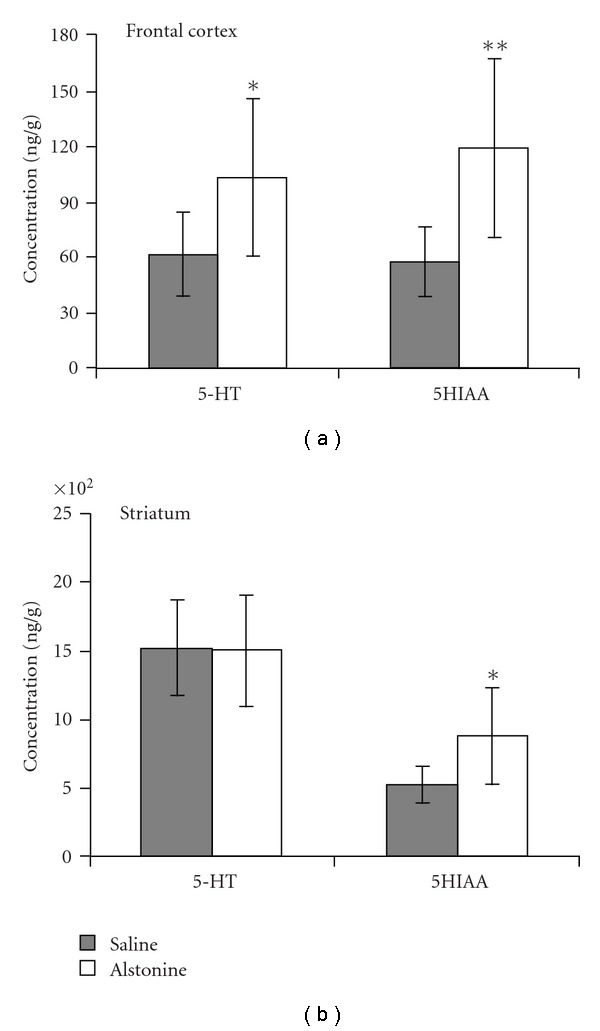
Serotonin (5-HT) and its metabolite (5HIAA) in mouse frontal cortex (a) and in striatum (b) Mean ± SD. *P < .05, **P < .01 when compared with saline, Independent *t*-test.

The effects of alstonine on brain amines are shown in Figures 2 and 3. DA levels were decreased (*t* = 4.96, *P* < .01) in frontal cortex (Figure 2(a)), with a concomitant increase in DOPAC (*t* = −2.22, *P* < .05) and no change in HVA. DOPAC levels were also increased (*t* = −3.62, *P* < .01) in the striatum (Figure 2(b)), without changes in DA or HVA. 5HT levels were increased only in the frontal cortex (*t* = −3.74, *P* < .05), whereas increases in 5-HIAA were seen in frontal cortex (*t* = −2.68, *P* < .01, Figure 3(a)) and striatum (*t* = −2.5, *P* < .05, Figure 3(b)).

**Figure 4:**
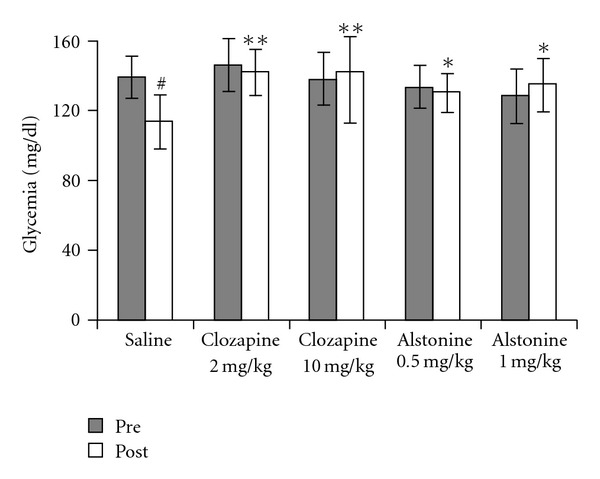
Effects of alstonine and clozapine on glucose levels. Mean ± SD ^*#*^ *P* < .01 compared with pre-drug, Paired *t*-test. *P < .05, **P < .01 compared with saline post-drug, ANOVA/Duncan.

**Figure 5:**
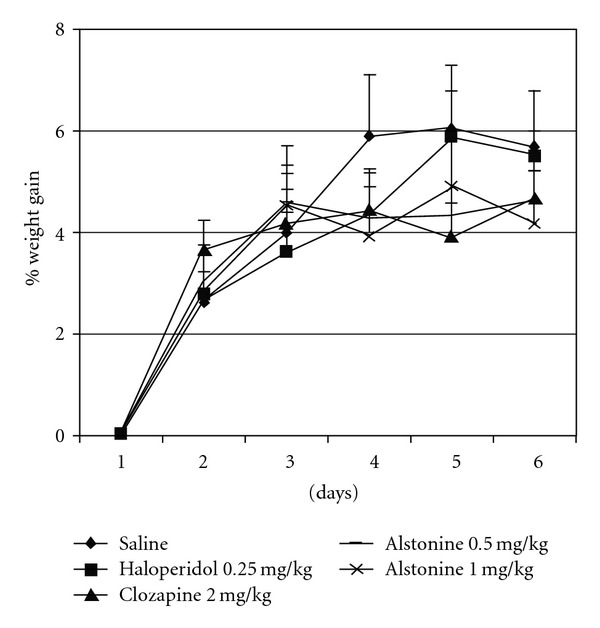
Body weight gain of mice treated with alstonine, haloperidol and clozapine. *n* = 8–10. Mean + SD, ANOVA with repeated measures/Duncan.

**Figure 6:**
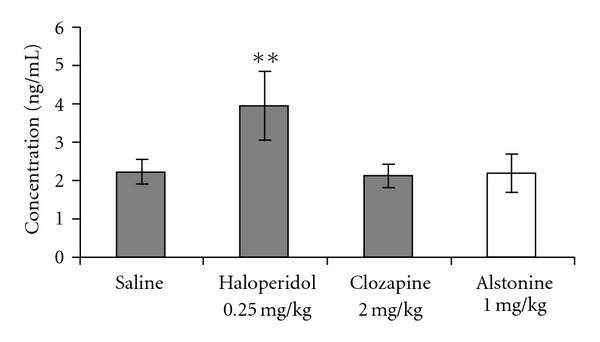
Effects of alstonine, haloperidol and clozapine on prolactin levels. Mean ± SD. **P < .01 when compared with saline. ANOVA/Duncan.

In control animals, as expected, a fasting-induced decrease in glucose levels was observed (*t* = 4.52, *P* < .01). The fasting-induced hypoglycaemia was absent with both clozapine and alstonine treatments (Figure 4). No differences in body weight gain were observed among treatment groups (Figure 5). Haloperidol (0.25 mg kg^−1^) markedly increased (F(3,23) = 12.78, *P* < .01) prolactin levels, whereas clozapine (2.0 mg kg^−1^) and alstonine (1.0 mg kg^−1^) did not (Figure 6).

## 4. Discussion

Improving the effectiveness of antipsychotics appears to require proper and specific modulation of the various DA pathways. For instance, lessened extrapyramidal symptoms and amelioration of negative symptoms observed with newer agents is thought to be consequent to differential effects on the striatum and frontal cortex, respectively [10].

Although the behavioural profile of alstonine appears to be closer to that of newer rather than older antipsychotics, it nevertheless differs from clozapine in its ability to reverse MK801-induced hyperlocomotion [15] and social deficit [16], and by lacking epileptogenic properties [22]. An unusual mechanism of action for alstonine is also here suggested by its effects on levels of DA in frontal cortex and striatum. Lack of changes in HVA levels suggests unchanged DA metabolism at the synaptic level, whereas increases in DOPAC levels suggests augmented intraneuronal catabolism.

DOPAC levels are accepted as a reliable index of intraneuronal DA degradation [23, 24], resulting from intracellular monoamine oxidase (MAO) activity. However, increases in DOPAC could also result from increased activity of the membrane transporter DAT, or even from the inhibition of the vesicular transporter VMAT2. All of these alternatives would eventually modulate DA availability at the synaptic level. The observed decrease in DA levels in the frontal cortex may not be significant behaviourally since we have recently shown that alstonine increased social interaction and prevented MK801 social withdrawal in mice [16], two accepted mouse models for behavioral equivalents of negative-like symptoms. Although only MAO or DAT inhibitor drugs are currently known, a direct effect of alstonine on these targets can not be ruled out at this point. Ongoing auto-radiography experiments will be useful to clarify this issue. In any case, a diminished DA availability at the synaptic level is consistent with alstonine's ability to counteract amphetamine-induced stereotypy and/or prevent lethality in amphetamine-treated grouped mice [14]. Although at this point it remains a matter of speculation, such an unusual neurochemical profile could open up innovative scenarios for antipsychotics and requires further scrutiny by specific experiments.

The HPLC analysis also shows that alstonine induced increases in 5HT levels in frontal cortex, and of 5HIAA in frontal cortex and striatum. The suggestion that alstonine acts as a 5HT_2A,C_ inverse agonist [15] is compatible with 5HT and 5HIAA increases, since pre-synaptic 5HT_2A,C_ receptors modulate 5HT release. 5HT modulation is considered to be central to the differential profile of newer antipsychotics, and dopamine modulation by serotonin is now generally accepted [11, 25–27]. 5HT_1A_ and 5HT_2A_ receptors stimulate DA release in the cortex, whereas 5HT_2C_ receptors may inhibit DA in the ventral tegmental area; it has been suggested that these combined properties could balance the DA system to the benefit of the management of schizophrenic symptoms [11].

It has been recognized that amino acid neurotransmitters may play a significant role in schizophrenia [28]. Although the anxiolytic effects of astonine were not modified by previous administration of picrotoxine (GABA_A_ antagonist), alstonine prevents the emergence of MK80-induced hyperlocomotion and social withdraw [15, 16]. Given that alstonine does not seem to interfere directly with [^3^H] glutamate release by cortical synaptosomes [29], the data is rather consistent with an indirect glutamate modulation, since 5-HT_2_ receptores are known to modulate NMDA glutamatergic transmission [30, 31]. Further studies are needed to better clarify the effects of alstonine on glutamate and GABA neurotransmission, and/or the role of these receptors on the amine changes induced by alstonine.

An extensive clinical trial comparing classical and atypical antipsychotics (known as CATIE) pointed to weight gain and hyperglycaemia as major causes of discontinuing therapy with the newer drugs [4]. Although treatment periods longer than those used here would have to be analyzed, neither alstonine nor clozapine or haloperidol altered mouse weight gain with doses effective in relevant behavioural mouse models. The precise mechanism by which antipsychotics alter glycemia is not completely clear. However, a number of candidates have been suggested such as H_1_ histamine receptors, 5HT_2C_ receptors, M_3_ muscarinic receptors, *α*_2_ adrenergic receptors and the inhibition of glucose transporters (GLUT) [7, 32]. Animal models of hyperglycemia appear to have a good correlation with clinically induced diabetes, and it is noticeable that not only atypical agents but also by some classic antipsychotic like chlorpromazine and perphenazine produce hyperglycemia in mice [32]. The fasting period used in this study induces a clear fall in the glycaemia levels of control animals, a drop absent in alstonine- and clozapine-treated mice. The data suggest that alstonine may share with atypical antipsychotics, such as clozapine, olanzapine and quetiapine, the unwanted changes in glucose metabolism. The exact mechanism by which such alterations occur requires explanation.

Relevant to adherence to treatment, a clear correlation exists between elevated PRL levels and DA D2 receptor occupancy by classical antipsychotics in the tuberoinfundibular pathway [2]. Although doses of antipsychotics used in rodents are usually much higher than those used in schizophrenic patients [7, 33, 34] there is a similar pattern of antipsychotic-induced prolactin release [35].

Different from haloperidol, alstonine and clozapine did not alter PRL levels, further confirming the atypical profile of the former and reinforcing its differential effects on the dopaminergic pathways.

In conclusion, this study reinforces the opinion that a comprehensive understanding of the neurochemical basis of alstonine's seemingly innovative profile as an antipsychotic may open new avenues to developing newer medications useful for the treatment of schizophrenic patients. Considering the gaps in the understanding of schizophrenia and the complexity of its neurochemical basis, this study underpins the value of traditional medical systems in unveiling original drugs.

## Funding

FINEP research grant “Rede Instituto Brasileiro de Neurociência (IBN-Net)" # 01.06.0842-00.
